# Emotion dysregulation in insomnia disorder: the possible role of psychiatric comorbidity

**DOI:** 10.3389/frsle.2024.1383552

**Published:** 2024-05-30

**Authors:** Markus Jansson-Fröjmark, Samiul Hossain

**Affiliations:** ^1^Department of Clinical Neuroscience, Centre for Psychiatry Research, Karolinska Institutet, Stockholm, Sweden; ^2^School of Psychology, Murdoch University, Murdoch, WA, Australia

**Keywords:** insomnia, emotion regulation, worry, beliefs, monitoring, safety behavior

## Abstract

**Aim:**

The purpose of this study was to investigate the link between emotion dysregulation and insomnia disorder as well as the possible role of psychiatric comorbidity on the association. More specifically, the aim was to examine whether the elevations in emotion dysregulation in insomnia are dependent on co-occurring psychiatric comorbidity, in this study defined as anxiety disorders and/or major depression.

**Methods:**

Four diagnostically differing groups with 25 participants in each were recruited: normal sleep, normal sleep with psychiatric comorbidity, insomnia disorder, and insomnia disorder with psychiatric comorbidity. The 100 study participants completed self-report scales and items assessing socio-demographic parameters, symptoms of insomnia, anxiety, and depression, generic emotion regulation, and insomnia-specific emotion regulation.

**Results:**

Concerning generic emotion regulation, the results showed that psychiatric comorbidity, but not insomnia, was associated with elevations in generic emotion dysregulation. Psychiatric comorbidity was distinctly related to elevations in non-acceptance, goals, and impulse domains (d = 1.09–1.22). Regarding insomnia-specific emotion regulation, the findings demonstrated that insomnia, with or without psychiatric comorbidity, was related to heightened use of insomnia-associated emotion dysregulation strategies. Insomnia was uniquely associated with elevated unhelpful beliefs about sleep and safety behaviors (d = 1.00–1.04).

**Conclusion:**

The current findings support the notion that insomnia is associated with specific, but not generic, emotion dysregulation strategies. These results have relevance for the conceptualization of the role of emotion dysregulation in insomnia and the clinical management of insomnia.

## Introduction

Insomnia, a common form of sleep disorder, is characterized by difficulties in falling asleep, frequent unintentional waking up during the night, inability to return to sleep after waking, and early morning waking, as well as associated daytime symptoms (American Psychiatric Association, 2013). Insomnia has been hypothesized to be characterized by emotion dysregulation (Riemann et al., 2022; Meneo et al., 2023). Emotion dysregulation is defined as difficulties in being aware of, accepting of, and understanding emotions (Gratz and Roemer, 2004). Emotion dysregulation can be associated with insomnia at two levels; an individual with insomnia may experience insomnia-specific emotion dysregulation, and generic emotion dysregulation compared to a good sleeper (Harvey, 2002; Galbiati et al., 2020; Tang et al., 2023). The first form of emotion dysregulation is disorder-specific and involves distinct strategies to relate to emotions, e.g., using worry in bed to manage an anxiety-provoking thought or employing monitoring/attention to detect sleep-related threats in the sleep environment that might trigger negative emotions. The second type of emotion dysregulation is universal and does not have to be associated with a specific disorder and may include strategies such as non-acceptance of emotional responses, difficulties with impulse control, and lack of emotional awareness.

Based on theory and preliminary evidence, insomnia appears to be characterized by heightened emotion dysregulation. Several conceptualizations of insomnia include emotion dysregulation as part of the framework to understand the interplay between insomnia and emotion dysregulation (Baglioni et al., 2010). Tentatively, sleep loss (acute or chronic) has been suggested to impair the ability to use emotion regulation strategies in a functional or flexible manner (Cerolini et al., 2015). During the past decades, there has been an even stronger focus in theory-building and empirical work to link mental disorders and emotion dysregulation. A review by Aldao et al. (2010) showed that across mental disorders, emotion dysregulation strategies appear to be elevated and maintaining factors for psychopathology. Of the mental disorders, anxiety and depression seem to be the disorders most strongly associated with emotion dysregulation, particularly so with the use of avoidance, rumination, and suppression strategies.

Emotion dysregulation, insomnia, and mental disorders share a complex and intricate relationship, as poor sleep may lead to inadequately regulated emotions, which may influence anxiety and depression, and these symptoms may further influence insomnia severity (Meneo et al., 2023). Likewise, comorbid anxiety and depressive disorders may reduce treatment efficacy for insomnia when treatment is delivered consisting only of parts of cognitive behavioral therapy for insomnia (Bélanger et al., 2016). Therefore, it is plausible that the association between emotion dysregulation and insomnia can be influenced by comorbid anxiety and depressive disorders. Thus, emotion dysregulation, insomnia, and comorbid mental disorders are intertwined with each other (Garland et al., 2018). Nevertheless, these constructs have nearly always been studied one by one or two by two, limiting our understanding of the intricate nature of the relationship between emotion dysregulation, insomnia, and comorbid anxiety and depression (Fairholme et al., 2013). In one of the few published studies with American adults, it was reported that when the presence of comorbid anxiety and depressive symptoms were statistically controlled, the association between emotion dysregulation and insomnia became non-significant (Gruber et al., 2008). These findings were later replicated in similar groups; anxiety and depression severity increased insomnia severity only in persons with high emotion dysregulation, but not in persons with low emotion regulation difficulties (Fairholme et al., 2013).

Exploring only anxiety as a mental disorder, studies have observed that anxiety severity moderates the association between emotion dysregulation and insomnia so that anxiety severity increases insomnia severity only in those experiencing high levels of emotion dysregulation (Kirwan et al., 2017; Wołyńczyk-Gmaj et al., 2022). Similarly, in a comparison between American adult participants with current, remitted depressive disorders, and healthy controls, it was found that emotion dysregulation moderated the association between sleep disturbance and depression (O'Leary et al., 2017). Taken together, it is plausible that comorbid anxiety and depression may influence the association between insomnia and emotion dysregulation so that the elevated emotion dysregulation observed in previous studies might be explained by psychiatric comorbidity.

### Present study

Our purpose was to investigate the association between emotion dysregulation and insomnia. More specifically, the aim was to explore whether the elevated emotion dysregulation in insomnia observed in previous research is due to psychiatric comorbidity. To that end, four distinct diagnostic groups were used: normal sleep, normal sleep with psychiatric comorbidity, insomnia disorder, and insomnia disorder with psychiatric comorbidity. Emotion dysregulation was explored using both generic emotion dysregulation measures and insomnia-specific emotion dysregulation assessments. The hypothesis was that psychiatric comorbidity would be associated with generic emotion dysregulation and insomnia with insomnia-specific emotion dysregulation.

## Methods

### Study participants

Participants were recruited through newspaper advertisements in Sweden informing about the possibility to take part in a study on sleep and emotions. All interested candidates were screened in two steps. The insomnia criteria used for inclusion and exclusion in the screening phases were based on research diagnostic criteria (Edinger et al., 2004b), the Diagnostic and Statistical Manual of mental disorders (DSM-5) (American Psychiatric Association, 2013), and validated, quantitative cutoffs (Lichstein et al., 2003; Morin et al., 2011). In the first screening step, candidates were asked to answer questions on a secure web application. The web-administered questions focused on socio-demographic parameters. It was required that the individuals were 18 years or older to participate. At the first step, all candidates also completed the Insomnia Severity Index (ISI) to be used as a total score in the screening process (Bastien et al., 2001), the Bergen Insomnia Scale (BIS) to be employed as a tool to determine the frequency (i.e., number of days) of nighttime insomnia symptoms (Pallesen, 2008), a question of whether they thought that they had experienced sleep disturbance during the past 3 months (yes/no), four items to assess sleep difficulties (sleep onset latency, wake after sleep onset, and early morning awakening, and total sleep time) during the past week, and one item from the Montgomery and Asberg Depression Rating Scale-Self report (MADRS-S) (Montgomery and Åsberg, 1979) to assess suicide risk. Finally, the SLEEP-50 (Spoormaker et al., 2005) was used as a screening tool to identify candidates with primary sleep disorders other than insomnia. Participants who exceeded cutoffs on the SLEEP-50 for the following primary sleep disorders were excluded: sleep apnea, narcolepsy, restless legs syndrome or periodic limb movement disorder, circadian rhythm disorder, sleepwalking, nightmares, and hypersomnia. In all, 11.9% (*n* = 21) of the 177 individuals at the first step were excluded due to exceeding SLEEP-50 cutoffs. None of the participants were excluded due to suicide risk, operationalized as 4 points (i.e., high suicidal ideation) or more on item 9 in MADRS-S.

The 156 candidates that met the criteria in the first step (see above) were then screened via telephone interview by the first author in the second screening step. Despite repeated attempts to reach the 156 individuals for a telephone interview, four of the candidates did not respond and were therefore excluded. As a result, 152 individuals were interviewed using two semi-structured interview formats, the Duke Structured Interview for Sleep Disorders (DSISD) and the Mini International Neuropsychiatric Interview (MINI) (Sheehan et al., 1998; Edinger et al., 2004a), to document sleep and mental disorders.

The following criteria had to be met to be included in the *normal sleep* group as assessed with the ISI, BIS, DSISD, and MINI (Edinger et al., 2004b): (a) ISI score below 10 points, (b) no early, middle or late insomnia according to the BIS, i.e. 0–1 days per week during the past month, (c) no complaints of sleep difficulties or associated daytime symptoms (a “0” response on all ISI items), (d) regular bedtimes and rising times (defined as ± 30 min deviation during a typical week in the past month, (e) no sleep-disruptive medical or mental disorder, and (f) no primary sleep disorder according to the DSISD. Criterion d was added to reduce the risk of including participants with occasional insomnia symptoms triggered by irregular sleep schedules and ensuing changes in sleep pressure. *Insomnia disorder* was operationalized as follows: (a) ISI score above 10 points, (b) early, middle, or late insomnia according to the BIS, i.e. 3 days per week or more during the past month, (c) complaints of sleep difficulties and associated daytime symptoms, (d) no sleep-disruptive medical or mental disorder or substance use, and (f) no primary sleep disorder other than insomnia according to the DSISD (sleep apnea, restless legs syndrome, periodic limb movement disorder, circadian rhythm disorder, and parasomnias). Psychiatric comorbidity was assessed with the MINI. The criteria for the following mental disorders had to be met to be included in one of the two *psychiatric comorbidity* groups: generalized anxiety disorder, major depression, panic disorder (with or without agoraphobia), and social anxiety disorder. Participants meeting the criteria for other mental disorders in the MINI were excluded. In all, 52 candidates were excluded during the telephone screening phase [due to other sleep disorders (*n* = 7), other mental disorders (*n* = 14), or a combination of sleep and insomnia criteria (*n* = 31)].

The final sample consisted of 100 participants, 25 normal sleepers, 25 normal sleepers with psychiatric comorbidity (i.e., anxiety and/or depression), 25 insomnia disorder, 25 insomnia disorder with psychiatric comorbidity (i.e., anxiety and/or depression). The study participants completed the remaining study variables (i.e., generic and insomnia-specific emotion dysregulation measures) on a secure web application after being included in the study. After completing the self-report instruments, each participant was compensated with a gift certificate (worth ~20 USD).

### Measures

The participants completed questions concerning socio-demographic parameters [i.e., age, gender, civil status (married or partner vs. no partner), occupational status (employment or educational activities vs. unemployment, retirement or on sick leave), educational level (college or university vs. high school or compulsory school), and birthplace (Sweden/not Sweden)].

#### Sleep and insomnia

Two self-report scales were used to assess insomnia symptoms: ISI (Bastien et al., 2001) and BIS (Pallesen, 2008). The Cronbach's alphas for the ISI and BIS were α = 0.83 and α = 0.81. Also, three items were employed to assess insomnia-characteristic sleep difficulties (sleep onset latency, wake after sleep onset, and early morning awakening) during the past week. The response alternatives for the three items were: < 15 min, 16–30 min, 31–60 min, > 60 min. The participants were also asked to estimate total sleep time during the past week (response alternatives: < 6 h, 6–7 h, 7–8 h, 8–9 h, 9–10 h, > 10 h). Also, the SLEEP-50 (Spoormaker et al., 2005) was employed to identify primary sleep disorders (sleep apnea, narcolepsy, restless legs syndrome or periodic limb movement disorder, circadian rhythm disorder, sleepwalking, nightmares, and hypersomnia). The SLEEP-50 has high internal consistency, test-retest correlation ranging between 0.65 and 0.89, and a factor structure in correspondence with the DSM-IV-TR sleep disorders. The sensitivity and specificity scores are acceptable (sensitivity: 0.67–1.00; specificity: 0.69–1.00). The agreement between clinical diagnoses and classification derived from the SLEEP-50 is substantial (kappa = 0.77) (Landis and Koch, 1977). The participants were asked to rate to what extent the items have been applicable during the past month (1 = not at all, 4 = very much).

#### Depression, suicide risk, and anxiety

The Patient Health Questionnaire (PHQ-9; Kroenke et al., 2001) was used to assess depression. Nine items are rated on a 4-point Likert scale (0 = not at all; 3 = nearly every day) corresponding to the DSM-IV criteria for depression. The tenth item assesses functional level (“How difficult have these problems made it for you to do your work, take care of things at home, or get along with other people?”). A PHQ-9 score of ≥10 has shown a sensitivity and specificity of 88% for capturing major depression. The internal consistency of the PHQ-9 in the present sample was high (α = 0.88). As part of the screening, item 9 from the MADRS-S (Montgomery and Asberg, 1979) was used to assess suicide risk. The Generalized Anxiety Disorder Screener (GAD-7; Spitzer et al., 2006) consists of seven items assessing anxiety-related symptoms during the past 2 weeks. The GAD-7 is rated on a 4-point Likert scale ranging from 1 (not at all) to 4 (nearly every day). The GAD-7 is associated with satisfactory reliability (Cronbach's α = 0.89–0.94) and convergent validity (Löwe et al., 2008; Mills et al., 2014). A GAD-7 score of >10 has been reported to suggest an anxiety disorder (Löwe et al., 2008). The internal consistency of the scale in the present sample was excellent (α = 0.91).

#### Generic emotion regulation

The Difficulties in Emotion Regulation Scale (DERS) was used to assess generic emotion regulation (Gratz and Roemer, 2004). The DERS is a multidimensional index of emotion regulation and contains six subscales with 36 items: non-acceptance of emotional responses (non-acceptance), difficulties engaging in goal-directed behavior (goals), impulse control difficulties (impulse), lack of emotional awareness (awareness), limited access to emotion regulation strategies (strategies), and lack of emotional clarity (clarity). Participants are asked to indicate how often the items apply to themselves, with responses ranging from 1 to 5, where 1 is almost never (0–10%), 2 sometimes (11–35%), 3 about half the time (36–65%), 4 most of the time (66–90%), and 5 almost always (91–100%). The DERS items were recoded so that higher scores in every case indicated greater difficulties in emotion regulation (i.e., greater emotion dysregulation). The internal consistency of the DERS and its subscales is high (α > 0.80). Analyses over time have shown that the test-retest reliability of the DERS and its subscales is adequate or good (Gratz and Roemer, 2004). The internal consistency of the total DERS using this study's sample was high at α = 0.84. The internal consistencies for the six subscales were between α = 0.76 (awareness) and α = 0.89 (goals).

#### Insomnia-specific emotion regulation

Four self-report instruments were used to assess insomnia-specific emotion regulation. The Anxiety and Preoccupation about Sleep Questionnaire was used to assess sleep-related worry (APSQ; Tang and Harvey, 2004; Jansson-Fröjmark et al., 2011). The response alternatives for each of the 10 items were 1 (strongly disagree) to 10 (strongly agree). In the current sample, the internal consistency was α = 0.91. To assess sleep-related beliefs, the Dysfunctional Beliefs and Attitudes about Sleep scale was used (DBAS-16) (Morin et al., 2007). The DBAS-16 contains 16 items with response alternatives from 0 (strongly disagree) to 10 (strongly agree). Based on the current sample, the internal consistency was α = 0.85. The Sleep Associated Monitoring Index was employed as an index of monitoring/attentional bias in insomnia (SAMI; Semler and Harvey, 2004). The number of items in the SAMI is 30 with response alternatives from 1 (not at all) to 5 (all the time). The internal consistency for the SAMI in the present sample was α = 0.81. The Sleep-Related Behaviors Questionnaire was used to assess safety behaviors in the context of insomnia (SRBQ; Ree and Harvey, 2004). The SRBQ assesses behaviors that are intended to improve fatigue, suppress thoughts, and improve sleep. The response alternatives for the 32 items were from 0 (almost never) to 4 (almost always). Based on the current sample, the internal consistency was α = 0.89.

### Statistical analysis

Chi-square test and ANOVA with *post-hoc* test were used to compare the four groups concerning socio-demographic parameters, sleep difficulties, insomnia severity, anxiety disorders, mood disorders, PHQ-9, and GAD-7. The main analyses employed ANOVA with *post-hoc* tests to examine group-wise differences on the DERS subscales, APSQ, DBAS-16, SAMI, and SRBQ. To control for the number of t-tests performed as *post-hoc* test, Bonferroni correction was carried out by dividing the conventional *p*-value (0.05) by the number of possible *t-*tests per emotion regulation strategy (6). The Bonferroni corrected *p*-value used in this study was thus p < 0.0083. Each significant group difference was followed by calculating Cohen's d to index the magnitude of the difference. Cohen (2013) proposed a 3-fold classification of effect sizes: small (0.20–0.49), medium (0.50–0.79), and large (0.80 and above).

## Results

### Socio-demographic parameters across the four groups

The descriptive statistics for the participants are displayed in Table 1. The age range for the participants was 19–69 years. As can be seen in the table, the four groups did not significantly differ from one another in terms of socio-demographic parameters.

**Table 1:** Socio-demographic parameters across the four groups.

	**NS**	**NS + PC**	**ID**	**ID + PC**	**F/χ^2^**
Age [M (SD)]	42.8 (15.7)	39.5 (15.7)	42.1 (15.3)	48.7 (12.8)	1.69
Gender (female)	48.0%	68.0%	68.0%	56.0%	3.00
Civil status (married or partner)^a^	84.0%	76.0%	68.0%	68.0%	2.29
Occupational status (work or student)^b^	88.0%	72.0%	92.0%	68.0%	6.50
Educational level (college or university)^c^	72.0%	68.0%	52.0%	64.0%	2.43
Birthplace (born in Sweden)^d^	92%	92%	92%	84%	1.33

All analyses are based on the full sample (*N* = 100). ID, insomnia disorder; ID + PC, insomnia disorder with psychiatric comorbidity; NS, normal sleepers; NS + PC, normal sleepers with psychiatric comorbidity. ^a^Compared with no partner. ^b^Compared with unemployment, retirement, or on sick-leave. ^c^Compared with compulsory school or high school. ^d^Cohen's d. Compared with born outside of Sweden.

### Sleep parameters, insomnia severity, mental disorders, and level of depression and anxiety across the four groups

As is displayed in Table 2, there were significant differences between the four groups concerning sleep parameters and insomnia severity; overall, the two groups with insomnia disorder consisted of significantly more participants exceeding cutoffs for clinical sleep onset latency, wake after sleep onset, and early morning awakenings, as well as significantly higher insomnia severity, relative to the two groups with normal sleep. Concerning total sleep time, there were significantly more individuals in the two groups with insomnia disorder, relative to normal sleepers, that slept shorter than 6 h per night, and significantly more participants in the insomnia disorder and psychiatric comorbidity compared to the normal sleep and psychiatric comorbidity group. Between 44% and 48% met the criteria for an anxiety disorder in the two psychiatric comorbidity groups (24% GAD, 14% PD, and 8% SAD). Between 52% and 56% fulfilled the criteria for major depression in the two psychiatric comorbidity groups. The two groups with psychiatric conditions scored significantly higher on the anxiety and depression symptoms scales, relative to the groups without mental conditions.

**Table 2:** Sleep parameters, insomnia severity, mental disorders, and levels of depression and anxiety across the four groups.

	**NS**	**NS + PC**	**ID**	**ID + PC**	**F/χ^2^**	**Significant group differences**
Sleep onset latency (>31 min)	4.0%	8.0%	36.0%	44.0%	16.88^*^	ID & ID + PC > NS & NS + PC
Wake after sleep onset (>31 min)	8.0%	16.0%	52.0%	68.0%	26.74^*^	ID & ID + PC > NS & NS + PC
Early morning awakening (>31 min)	12.0%	16.0%	40.0%	72.0%	25.10^*^	ID & ID + PC > NS & NS + PC
Total sleep time (< 6 hours)	16.0%	24.0%	48.0%	56.0%	11.81^*^	ID & ID + PC > NS ID + PC > NS + PC
ISI	3.0 (1.8)	4.1 (1.7)	13.0 (2.2)	15.8 (3.3)	189.05^**^	ID & ID + PC > NS & NS + PC ID + PC > ID
Anxiety disorders	0%	48.0%	0%	44.0%	29.98^**^	NS + PC & ID + PC > NS & ID
Mood disorders	0%	52.0%	0%	56.0%	77.00^**^	NS + PC & ID + PC > NS & ID
PHQ-9	3.1 (1.9)	12.1 (4.2)	8.6 (2.9)	13.4 (4.3)	44.02^**^	NS + PC & ID & ID + PC > NS NS + PC & ID + PC > ID
GAD-7	7.8 (5.1)	13.4 (4.6)	9.3 (5.2)	13.6 (4.2)	9.31^**^	NS + PC & ID + PC > NS & ID

All analyses are based on the full sample (*N* = 100). ID, insomnia disorder; ID + PC, insomnia disorder with psychiatric comorbidity; NS, normal sleepers; NS + PC, normal sleepers with psychiatric comorbidity. ^*^p < 0.05, ^**^p < 0.01.

### Generic emotion dysregulation in insomnia disorder

Six one-way ANOVAs were executed to examine potential differences in the generic emotion regulation subscales between the four groups. The results are displayed in Table 3. *Post-hoc* tests showed that significant group differences emerged for three of the DERS subscales. The group diagnosed with insomnia disorder and psychiatric comorbidity scored significantly higher and with large effect sizes, relative to the normal sleep group, on the non-acceptance subscale. Relative to those with insomnia disorder only, the group with insomnia disorder and psychiatric comorbidity scored significantly higher and with large effect sizes on the goals and impulse subscales. Finally, normal sleepers with psychiatric comorbidity reported a significantly higher level and with large effect sizes on the impulse subscale, relative to those with insomnia disorder only.

**Table 3:** Generic emotion dysregulation in insomnia disorder: relative to normal sleep and psychiatric comorbidity.

	**NS**	**NS + PC**	**ID**	**ID + PC**	**F (p)**	**Significant group differences and effect sizes**
Non-acceptance (DERS)	8.6 (2.8)	11.9 (5.4)	9.2 (4.3)	13.6 (6.4)	5.52 (0.002)	ID + PC > NS: d = 1.09
Goals (DERS)	11.5 (5.6)	13.6 (5.4)	10.3 (3.6)	15.7 (6.0)	5.18 (0.002)	ID + PC > ID: d = 1.13
Impulse (DERS)	9.0 (4.7)	11.5 (5.5)	7.3 (2.6)	12.5 (5.9)	5.94 (0.001)	ID + PC > ID: d = 1.22 NS + PC > ID: d = 1.04
Awareness (DERS)	18.5 (6.5)	18.5 (5.9)	19.3 (6.9)	19.1 (6.0)	0.11 (0.952)	
Strategies (DERS)	10.4 (4.3)	15.4 (6.7)	9.8 (5.8)	15.7 (8.6)	5.85 (0.001)	
Clarity (DERS)	8.8 (2.6)	10.2 (3.4)	7.7 (3.7)	9.8 (3.7)	2.73 (0.048)	

All analyses are based on the full sample (*N* = 100). The *p*-value was adjusted to 0.0083 per emotion regulation strategy (0.05 divided by 6 *post-hoc* t-tests). Degrees of freedom for the analyses: 3. DERS, Difficulties in Emotion Regulation Scale; ID, insomnia disorder; ID + PC, insomnia disorder with psychiatric comorbidity; NS, normal sleepers; NS + PC, normal sleepers with psychiatric comorbidity.

Subsequent subgroup analyses examined whether the type of psychiatric comorbidity (i.e., anxiety disorders vs. major depression) had a role in the association between group status, as studied above, and generic emotion dysregulation. The descriptive statistics and results from ANOVA analyses are displayed in the Supplementary Table S1. While there were no significant group differences focusing solely on those with major depression, there were significant findings on three generic emotion regulation subscales (goals, impulse, and strategies), concentrating only on those with anxiety disorders.

### Insomnia-specific emotion dysregulation in insomnia disorder

Four one-way ANOVAs were executed to investigate potential differences in insomnia-specific worry, sleep-related beliefs, monitoring/attentional bias, and safety behaviors between the four groups (see Table 4). Following *post-hoc* tests, significant group differences emerged for all four measures. The group diagnosed with insomnia disorder and psychiatric comorbidity scored significantly higher and with large effect sizes, relative to normal sleepers, on all four scales. Also, those with insomnia disorder only, relative to normal sleepers, reported a significantly higher level and with large effect sizes of unhelpful beliefs and safety behaviors. Relative to normal sleepers, the group with both normal sleep and psychiatric comorbidity scored significantly higher and with large effect sizes on the safety behavior measure.

**Table 4:** Insomnia-specific emotion dysregulation in insomnia disorder: relative to normal sleep and psychiatric comorbidity.

	**NS**	**NS + PC**	**ID**	**ID + PC**	**F (p)**	**Significant group differences and effect sizes**
Worry (APSQ)	25.7 (9.2)	33.8 (15.8)	37.8 (15.9)	51.0 (20.6)	11.03 (< 0.001)	ID + PC > NS: d = 1.70
Unhelpful beliefs about sleep (DBAS-16)	4.3 (1.2)	5.1 (1.3)	5.5 (1.1)	6.2 (1.6)	9.52 (< 0.001)	ID + PC > NS: d = 1.36 ID > NS: d = 1.04
Selective attention and monitoring (SAMI)	55.8 (13.4)	68.9 (18.7)	70.4 (21.6)	77.4 (17.4)	6.25 (0.001)	ID + PC > NS: d = 1.40
Safety behaviors (SRBQ)	14.3 (10.4)	28.7 (12.9)	27.3 (15.7)	43.3 (20.5)	14.36 (< 0.001)	ID + PC > NS: d = 1.88 ID > NS: d = 1.00 NS + PC > NS: d = 1.24

All analyses are based on the full sample (*N* = 100). The *p*-value was adjusted to 0.0083 per emotion regulation strategy (0.05 divided by 6 *post-hoc* t-tests). Degrees of freedom for the analyses: 3. APSQ, Anxiety and Preoccupation about Sleep Questionnaire; DBAS-16, Dysfunctional Beliefs and Attitudes about Sleep (16-items version); ID, insomnia disorder; ID + PC, insomnia disorder with psychiatric comorbidity; NS, normal sleepers; NS + PC, normal sleepers with psychiatric comorbidity; SAMI, Sleep Associated Monitoring Index; SRBQ, Sleep-Related Behaviors Questionnaire.

Subsequent subgroup analyses examined whether psychiatric comorbidity condition (i.e., anxiety disorders vs. major depression) had a role in the association between group status and insomnia-specific emotion dysregulation. The descriptive statistics and results from ANOVA analyses can be seen in the Supplementary Table S2. There were significant group differences for both psychiatric comorbidity groups on all four insomnia-specific measures.

## Discussion

The purpose of this study was to examine the link between emotion dysregulation and insomnia disorder as well as the possible role of psychiatric comorbidity. Regarding generic emotion regulation, the findings imply that psychiatric comorbidity, but not insomnia, is associated with elevated generic emotion dysregulation. Turning to insomnia-specific emotion regulation, the results point toward the notion that insomnia, with or without psychiatric comorbidity, is related to heightened use of insomnia-associated emotion dysregulation strategies. These findings are in line with previous work on the role of disorder-specific strategies (Aldao et al., 2010).

Our finding that psychiatric comorbidity is associated with elevated generic emotion dysregulation is in line with previous results (e.g., Gruber et al., 2008; Aldao et al., 2010). Several results did, however, point to the possibility that insomnia is not characterized by generic emotion dysregulation. First, the fact that the insomnia group was not related to higher emotion dysregulation than normal sleepers was surprising (e.g., Gruber et al., 2008). Albeit not significant, the insomnia group even had lower scores than the normal sleep group on some generic emotion regulation subscales. Second, the two groups with psychiatric comorbidity reported higher generic emotion dysregulation on two generic emotion regulation subscales (i.e., goals and impulse), relative to the insomnia group. In all, these findings suggest that insomnia disorder is not associated with generic emotion dysregulation. A recent case-series study, adding emotion regulation strategies to an insomnia-focused intervention, resulted in insomnia improvements but not in changes in emotion regulation strategies (Byrne et al., 2020). In turn, this implies tentatively that assessing and intervening upon generic emotion dysregulation will likely not be an effective route for those with insomnia disorder.

Insomnia was, however, clearly related to insomnia-specific emotion dysregulation. Insomnia was uniquely associated with significantly higher ratings on unhelpful beliefs about sleep and elevations in the use of insomnia-related safety behaviors. The fact that the insomnia group did not report higher insomnia-associated emotion dysregulation than normal sleepers with psychiatric comorbidity was, however, surprising. One possible interpretation of this finding is to regard psychiatric comorbidity in isolation as elevating the use of insomnia-specific emotion dysregulation strategies. This interpretation could be viewed in light of the transdiagnostic model (Harvey et al., 2004), in which psychological mechanisms (e.g., worry) are shared across mental disorders (e.g., insomnia and GAD). Overall, the insomnia group with psychiatric comorbidity had the most consistent elevations in insomnia-specific emotion dysregulation in comparison with the other groups. In all, these findings suggest that psychiatric comorbidity heightens the use of insomnia-specific emotion dysregulation strategies, above and beyond insomnia alone.

In theoretical terms, the current findings support the notion that insomnia is associated with insomnia-specific, but not generic, emotion dysregulation. These results support the cognitive model of insomnia (Harvey, 2002) in the sense that this model emphasizes insomnia-specific processes as maintaining insomnia. Also, the findings have heuristic value for models that propose that sleep disturbance is related to emotion regulation (e.g., Harvey, 2011); this notion seems valid for insomnia-specific processes, and not for generic emotion dysregulation strategies given the current study's findings. These findings support the idea of assessing and treating insomnia-related emotion dysregulation strategies when insomnia, with or without psychiatric comorbidity, is present in clinical practice. The insomnia-specific emotion dysregulation strategies examined in this study – worry, sleep-related beliefs, monitoring/attentional bias, and safety behaviors – are important factors in the cognitive model of insomnia, and the current findings might thus have relevance for cognitive therapy in particular (Harvey, 2002). At least three studies have so far shown that cognitive therapy for insomnia, which aims to reverse insomnia-specific processes, is effective in reducing insomnia symptomatology and associated psychiatric symptoms (Harvey et al., 2007, 2014; Sunnhed et al., 2019). It is, however, important to emphasize that the therapy with the strongest empirical support for insomnia – cognitive behavioral therapy for insomnia – is also likely to reverse the emotion dysregulation processes explored in the current study. The additional focus on generic emotion dysregulation strategies might lead to enhanced assessment and treatment among those with psychiatric comorbidity, but this health care approach is likely less successful for those with insomnia only.

The current study has a number of methodological limitations that are relevant for the interpretation of the findings and for future research. A first limitation is the self-selection procedure that was used, thereby paving way for uncertainty regarding the generalizability of the findings. A related limitation is also that the recruitment procedure (newspaper advertisements) might limit the generalizability to other groups, such as patients with insomnia disorder and psychiatric comorbidity in health care settings. Second, since psychiatric comorbidity was defined as meeting criteria for an anxiety disorder and/or depression in this study, generalizability to other conditions is unknown. A third limitation was that statistical power is a possible explanation for some of the non-significant findings. This relates evidently even more so regarding the subgroup analyses, which consisted of comparing 11–25 individuals across groups. A fourth limitation relates to the cross-sectional design of the investigation, making conclusions regarding cause-effect (i.e., between insomnia, psychiatric comorbidity, and emotion regulation) and third variables (e.g., genes and medications) not reasonable. Fifth, emotion regulation was assessed with self-report data, which only allowed us to assess emotional experience in the subjective domain. Sixth, based on that a portion of insomnia patients can be characterized as having sleep loss, which may have negative effects on mood and emotion regulation (e.g., Watling et al., 2017), an additional limitation is that no objective sleep assessments were used in the current study. Finally, a myriad of models have been developed to measure and explain emotion regulation and these models measured unique aspects of emotion regulation (Naragon-Gainey et al., 2017). As our study only focused on one model of emotion regulation, it may not capture all the aspects of emotion regulation and its association with insomnia.

Future research is needed to clarify a number of issues following this study. First, the generalizability of the current findings is uncertain, and future studies could recruit and randomly select participants from various health care settings and ensure that patients with comorbidities other than anxiety and depression are included. Future studies might also base their power estimations on the current study so that appropriate sample sizes are included. It is also important that other emotion regulation domains and non-subjective measures are explored in future research. Finally, of utmost importance is to investigate emotion dysregulation, insomnia disorder, and psychiatric comorbidity using a longitudinal approach. One way to go about this would be to measure the three areas repeatedly with self-report scales and non-experimentally explore the role of insomnia and psychiatric symptoms on changes in emotion regulation (both generic and insomnia-specific forms) over time. Building on the current study's findings, one could suspect that psychiatric symptoms would be stronger predictors of increases in emotion dysregulation than insomnia symptoms. With such an approach, one could also examine the role of emotion regulation in the development of insomnia and psychiatric symptoms. A different, more causative approach could be to investigate the reductions in emotion dysregulation that take place when insomnia interventions (e.g., cognitive behavioral therapy for insomnia) and psychiatric interventions (e.g., pharmacotherapy or psychosocial therapies) are compared against each other among patients with insomnia disorder and psychiatric comorbidity. Based on what was found in the current investigation, one could hypothesize that psychiatric interventions, relative to insomnia therapies, would have a stronger effect on emotion dysregulation.

The current study's findings demonstrate that generic emotion dysregulation is not enhanced in those with insomnia disorder, implying that broad emotion regulation strategies are less likely to play a role in the maintenance of insomnia. The fact that insomnia disorder was uniquely associated with several insomnia-specific emotion dysregulation strategies underscores the likelihood that insomnia is maintained by disorder-specific strategies to regulate emotions. Also, the findings emphasize the need to pinpoint insomnia-specific emotion dysregulation strategies when managing insomnia in clinical practice.

## Data availability statement

The datasets presented in this article are not readily available because the study does not have ethical permission to share data. Requests to access the datasets should be directed to markus.jansson-frojmark@ki.se.

## Ethics statement

The studies involving humans were approved by the Etikprövningsmyndigheten in Stockholm, Sweden. The studies were conducted in accordance with the local legislation and institutional requirements. The participants provided their written informed consent to participate in this study.

## Author contributions

MJ-F: Conceptualization, Data curation, Formal analysis, Investigation, Methodology, Project administration, Resources, Validation, Writing—original draft, Writing—review & editing. SH: Conceptualization, Writing—original draft, Writing—review & editing.
