# Supplementary material for: Emotion dysregulation in insomnia disorder: the possible role of psychiatric comorbidity
Source: Front Sleep. 2024 May 30;3:1383552. doi: 10.3389/frsle.2024.1383552 (PMC12713947; doi:10.3389/frsle.2024.1383552)
Supplement: Supplementary file 1 [file Table_1.docx]

Supplementary Table 1 (S1)

*Generic emotion dysregulation in insomnia disorder: Relative to normal sleep and psychiatric comorbidity (subgroup analysis on anxiety disorders and major depression separately)*

|  | NS  (n=25) | NS + AD  (n=12) | ID  (n=25) | ID + AD  (n=11) | F (p) | Significant group differences and effect sizes |
| --- | --- | --- | --- | --- | --- | --- |
| Non-acceptance (DERS) | 8.6 (2.8) | 12.8 (6.9) | 9.2 (4.3) | 12.8 (6.9) | 3.81 (.014) |  |
| Goals (DERS) | 11.5 (5.6) | 16.5 (4.7) | 10.3 (3.6) | 13.9 (5.0) | 5.19 (.003) | NS + AD > ID: d = 1.56 |
| Impulse (DERS) | 9.0 (4.7) | 14.8 (5.9) | 7.3 (2.6) | 11.5 (4.8) | 8.72 (<.001) | NS + AD > ID: d = 1.90 |
| Awareness (DERS) | 18.5 (6.5) | 22.3 (4.3) | 19.3 (6.9) | 17.2 (5.9) | 1.44 (.238) |  |
| Strategies (DERS) | 10.4 (4.3) | 18.3 (6.7) | 9.8 (5.8) | 15.3 (8.4) | 7.39 (<.001) | NS + AD > ID: d = 1.39 |
| Clarity (DERS) | 8.8 (2.6) | 9.6 (2.6) | 7.7 (3.7) | 9.3 (2.5) | 1.39 (.254) |  |
|  | NS  (n=25) | NS + MD  (n=13) | ID  (n=25) | ID + MD  (n=14) |  |  |
| Non-acceptance (DERS) | 8.6 (2.8) | 11.1 (5.4) | 9.2 (4.3) | 14.1 (6.2) | 5.13 (.003) |  |
| Goals (DERS) | 11.5 (5.6) | 11.0 (4.7) | 10.3 (3.6) | 17.1 (6.5) | 6.02 (.001) |  |
| Impulse (DERS) | 9.0 (4.7) | 8.5 (2.7) | 7.3 (2.6) | 13.3 (6.7) | 5.87 (.001) |  |
| Awareness (DERS) | 18.5 (6.5) | 15.0 (4.9) | 19.3 (6.9) | 20.6 (5.9) | 1.99 (.124) |  |
| Strategies (DERS) | 10.4 (4.3) | 12.6 (5.6) | 9.8 (5.8) | 16.0 (9.0) | 3.67 (.016) |  |
| Clarity (DERS) | 8.8 (2.6) | 10.8 (4.0) | 7.7 (3.7) | 10.2 (4.4) | 2.65 (.055) |  |

*Note*. All analyses are based on the full sample (N = 100). The p-value was adjusted to .0083 per emotion regulation strategy (.05 divided by 6 post hoc t-tests). Degrees of freedom for the analyses: 3. DERS = Difficulties in Emotion Regulation Scale, ID = insomnia disorder, ID + AD = insomnia disorder with anxiety disorder, ID + MD = insomnia disorder with major depression, NS = normal sleepers, NS + AD = normal sleepers with anxiety disorder, NS + MD = normal sleep with major depression.
