# Supplementary material for: Emotion dysregulation in insomnia disorder: the possible role of psychiatric comorbidity
Source: Front Sleep. 2024 May 30;3:1383552. doi: 10.3389/frsle.2024.1383552 (PMC12713947; doi:10.3389/frsle.2024.1383552)
Supplement: Supplementary file 2 [file Table_2.docx]

Supplementary Table 2 (S2)

*Insomnia-specific emotion dysregulation in insomnia disorder: Relative to normal sleep and psychiatric comorbidity (subgroup analysis on anxiety disorders and major depression separately)*

|  | NS  (n=25) | NS + AD  (n=12) | ID  (n=25) | ID + AD  (n=11) | F (p) | Significant group differences and effect sizes |
| --- | --- | --- | --- | --- | --- | --- |
| Worry (APSQ) | 25.7 (9.2) | 35.0 (17.2) | 37.8 (15.9) | 52.4 (22.4) | 7.98 (<.001) |  |
| Unhelpful beliefs about sleep (DBAS-16) | 4.3 (1.2) | 5.2 (1.3) | 5.5 (1.1) | 6.2 (0.9) | 9.14 (<.001) | ID + AD > NS: d = 1.81  ID > NS: d = 1.04 |
| Selective attention and monitoring (SAMI) | 55.8 (13.4) | 74.4 (17.9) | 70.4 (21.6) | 76.7 (11.9) | 5.74 (.001) | ID + AD > NS: d = 1.65 |
| Safety behaviors (SRBQ) | 14.3 (10.4) | 29.8 (12.9) | 27.3 (15.7) | 35.6 (15.8) | 7.67 (<.001) | ID > NS: d = 2.75 |
|  | NS  (n=25) | NS + MD  (n=13) | ID  (n=25) | ID + MD  (n=14) |  |  |
| Worry (APSQ) | 25.7 (9.2) | 32.6 (15.0) | 37.8 (15.9) | 49.9 (19.8) | 8.47 (<.001) | ID + MD > NS: d = 1.67 |
| Unhelpful beliefs about sleep (DBAS-16) | 4.3 (1.2) | 5.1 (1.4) | 5.5 (1.1) | 6.2 (2.1) | 6.68 (<.001) | ID > NS: d = 1.04 |
| Selective attention and monitoring (SAMI) | 55.8 (13.4) | 63.8 (18.6) | 70.4 (21.6) | 77.9 (21.2) | 4.90 (.004) |  |
| Safety behaviors (SRBQ) | 14.3 (10.4) | 27.7 (13.4) | 27.3 (15.7) | 48.7 (22.2) | 14.83 (<.001) | ID + MD > NS: d = 2.11  ID > NS: d = 1.00 |

*Note*. All analyses are based on the full sample (N = 100). The p-value was adjusted to .0083 per emotion regulation strategy (.05 divided by 6 post hoc t-tests). Degrees of freedom for the analyses: 3. APSQ = Anxiety and Preoccupation about Sleep Questionnaire, DBAS-16 = Dysfunctional Beliefs and Attitudes about Sleep (16-items version), ID = insomnia disorder, ID + AD = insomnia disorder with anxiety disorder, ID + MD = insomnia disorder with major depression, NS = normal sleepers, NS + AD = normal sleepers with anxiety disorder, NS + MD = normal sleep with major depression, SAMI = Sleep Associated Monitoring Index, SRBQ = Sleep-Related Behaviours Questionnaire.
